# Domain-specific self-perceptions of aging are associated with different gait patterns in older adults: a cross-sectional latent profile analysis

**DOI:** 10.1186/s12877-021-02320-9

**Published:** 2021-06-29

**Authors:** Anne Blawert, Sebastian Krumpoch, Ellen Freiberger, Susanne Wurm

**Affiliations:** 1 Department of Prevention Research and Social Medicine, Institute for Community Medicine, University Medicine Greifswald, Walther-Rathenau-Str. 48, 17475 Greifswald, Germany; 2 Institute for Biomedicine of Aging, Friedrich-Alexander-Universität Erlangen-Nürnberg, Nürnberg, Germany

**Keywords:** Self-perceptions of aging, Gait profile, Gait pattern, Older adults, Views on aging

## Abstract

**Background:**

Previous studies have pointed to the impact of self-perceptions of aging (SPA) on self-reported physical function in later life. However, less is known about associations of SPA with objectively measured physical function, especially gait. Research that examined other psychological variables and objectively measured gait has focused on single gait parameters such as gait speed, which seems to fall short for the complexity of this movement. Some approaches have proposed ways to identify gait patterns in specific patient groups, but not in community samples. Our goal was (a) to identify gait patterns based on a combination of important gait parameters in a community sample, and (b) to investigate differential associations of gain- and loss-related SPA with these gait patterns.

**Methods:**

The study used an electronic walkway to assess gait parameters of 150 community dwelling adults aged 71–93 years (61.0% women) at their usual and maximum gait speed. SPA were assessed with a questionnaire. We used latent profile analysis (LPA) to identify groups exhibiting distinct gait patterns and binary logistic regression to investigate associations of SPA with these groups, controlling for personality traits, number of illnesses, age, gender, and education. To compare overall function between groups, a t-test for scores in the Short Physical Performance Battery was used.

**Results:**

LPA revealed two distinct groups in both gait speed conditions. The fit group exhibited a stable, well-coordinated and faster gait pattern, while the functionally limited group’s gait pattern was less stable, less coordinated and slower. The odds of belonging to the functionally limited group were increased by loss-related SPA at usual gait speed, while the odds of belonging to the fit group were increased by gain-related SPA at individual maximum speed.

**Conclusions:**

The findings (a) suggest LPA as a useful approach to investigate complex gait patterns considering several gait parameters simultaneously, and (b) provide first evidence for differential associations of gain- and loss-related SPA with gait patterns at usual and maximum gait speed. Intervention studies addressing gait in older adults should additionally address gain-related views on aging.

## Background

### Self-perceptions of aging and physical function

In recent years, a large number of studies have provided broad evidence for the impact of self-perceptions of aging (SPA) on physical and mental health and even mortality [1, 2]. SPA refer to an individual’s overall expectation and attitude towards the aging process. Especially in old age, SPA also increasingly reflect a person’s own actual aging experiences [3]. SPA are multidirectional, which means a person can hold both positive or gain-related and negative or loss-related SPA at the same time. SPA are also multidimensional: they refer to different life domains, e.g. to health, self-development or social relationships. Positive SPA can refer to gains such as personal growth and development, having time to make new plans and being able to pursue new ideas. Negative, loss-related SPA usually refer to physical or social losses, such as physical decline, illness or loss of social embeddedness.

By now, there is abundant evidence for the importance of SPA for the way people actually grow older [3, 4]. For example, people with more positive SPA report better physical and mental health [5] and even live up to 7.5 years longer [1], while people with more negative SPA are at increased risk of physical decline or frailty [6, 7]. However, most findings in this context rely on self-report data for physical function obtained by measures like the Short Form Health Survey [8, 9] or the Health Scale for the Aged [10]. These might be subject to bias through social desirability or simply because some people over- or underestimate their actual physical performance, e.g. their ability to walk several blocks without a break. It is therefore important to accumulate additional knowledge about associations of SPA with objectively measured physical function.

So far, only few studies have addressed this question. In addition, we see a very limited range of outcomes in these studies—they either looked at composite measures of physical performance like the Short Physical Performance Battery [11] or at single gait parameters like gait speed [12].

### Gait as an indicator of physical function

Gait speed is a gait parameter that is relatively easy—albeit not trivial [13]—to measure and associated with a large range of health outcomes and has even been termed “the functional vital sign” [14]. Modern technological devices allow the detailed recording of a large range of gait parameters that give additional information on specific gait features over and beyond gait speed such as step-width-variability or walk ratio. As some gait parameters change with gait speed, recording walking at different gait speeds is recommended [15]. Participants complete the walk at their usual pace and at their individual maximum speed. This provides important indications of everyday functioning of older adults. Walking at a usual pace reflects everyday behavior, like the ability to go grocery shopping. Yet, when crossing a road at a traffic light, it is also important to be able to keep a stable gait while walking at increased speed. At maximum speed, limitations in walking capacity become more noticeable [15].

However, most studies have focused on the role of single gait parameters for e.g. incident disability [16] or compared single gait parameters between groups [17]. Since walking is a complex task that requires coordination of diverse motion sequences, looking at gait patterns as a combination of several different gait parameters might be of additional value. Some studies proposed methods to form gait patterns, e.g. through cluster analysis or latent profile analysis. However, these studies were interested in identifying gait patterns associated with specific illnesses [18, 19], and not among community samples of older adults. Furthermore, studies on psychological correlates of gait mostly focused on the role of proximal variables like attentional influences [20], fear of falling [21], and trait conscious movement processing [22, 23] that are directly relevant for gait. However, several studies also provided evidence for associations of personality traits as more distal psychological variables with objectively measured gait in older adults [24, 25]. In this study, we aimed at investigating associations with gait patterns of another distal variable that has been related to physical function, namely, self-perceptions of aging.

### Aims and hypotheses

Taken together, as to our knowledge, this is the first study, which regards the complexity of gait while taking multidimensional and multidirectional aspects of SPA into account. We aimed at investigating associations of loss- and gain-related SPA, which refer to two different life domains, with gait patterns in a community sample of older adults.

To reach this goal, we first explored gait patterns in a community sample, and then—in a second step—investigated associations of SPA with these gait patterns. Based on previous research on SPA and physical function, we developed two specific hypotheses for associations of loss- and gain-related domains of SPA with gait patterns.

First, the loss-related domain of SPA physical losses refers to the notion of aging as associated with decline in health, fitness and vitality. This SPA domain is closely connected to the experience of actual physical losses in a reciprocal manner. On the one hand, many people who associated their own aging with physical losses report worse physical function [9] over time than those who do not have this view on aging, suggesting a longitudinal impact of this SPA domain on health. On the other hand, individuals often experience age-related physical decline and accumulating illnesses and then tend to attribute this to aging [26], resulting in the perception of aging as associated with physical losses. Since stronger physical limitations are already noticeable in everyday behavior and not only when investing effort, we hypothesized:

- H1: Higher SPA physical losses is associated with a higher likelihood to exhibit a functionally limited gait pattern when walking at usual gait speed.

Second, the gain-related domain of SPA ongoing development is a more motivational facet of SPA that refers to aging being associated with developing new plans, learning new things and following ideas. Thus, a person who associates their aging with ongoing development likely has certain resources available. For instance, gain-related SPA were related to tenacious goal pursuit in a previous study beyond a range of control variables [27]. Thus, a gain-related view on aging seems to be associated with a readiness to invest effort in reaching a goal. In addition, previous research found SPA ongoing development to buffer the negative effect of precariousness on health and well-being [28] and promote health behavior [29]. Positive SPA are also associated with a lower rate of overnight hospitalizations after 4 years [30], pointing to better health in older adults with positive SPA. Furthermore, the association of positive SPA and better self-reported physical function in late life is mediated by self-efficacy [8]: This means that positive SPA foster a person’s ability to cope successfully with aging-related challenges, which is then later reflected in better self-reported, and, presumably, also objective physical function. However, it might also be the case that older adults who experience little physical limitations are more likely to perceive their aging as associated with ongoing development, since they can focus on making new plans instead of being troubled by illnesses. Since our sample of older adults was very healthy and fit, we expected the domain of ongoing development to only be associated with a more favorable gait pattern at maximum gait speed. Walking at maximum speed requires effort and motivates participants to invest resources; thus, a distal psychological variable like SPA ongoing development might only become visible in this rather challenging condition.

- H2: Higher SPA ongoing development is associated with a higher likelihood of exhibiting a more favorable gait pattern when walking at individual maximum gait speed.

## Methods

### Participants and procedure

The study assessments took place in the city of Nuremberg in southeastern Germany. In total, 150 participants (61.0% women) were recruited via an existing address pool and distribution of additional flyers. Inclusion criteria were (1) an age of 70 years or older, (2) living independently, (3) ability to walk 10 m without a wheeled walker and (4) ability to understand and follow the test protocol. Participants were excluded if they reported serious orthopedical and/or neurological disorders that impeded walking. Participants needed to be able to come to the research institute were the assessment took place by themselves. At the study site, they completed several walks on an electronic walkway in a well-lit hallway and answered a structured personal interview that lasted approximately 120–240 min as part of a larger project. The participants also completed the Montreal Cognitive Assessment. The FAU Ethical Committee approved of the study (Reference number: 43_19B) and all participants gave their written informed consent.

### Measures

#### Gait parameters

The GAITRite system is a 10-m-long walkway with embedded pressure sensors (Gold walkway, 972 cm long, active electronic surface area 792 × 61 cm, total 29,952 pressure sensors, scanning frequency 60 Hz, GAITRite, CIR Systems Inc., Franklin, USA). Previous studies have shown that the GAITRite system is a valid and reliable instrument to measure gait parameters [31, 32]. The active sensor area of the walkway is 8 m long. Two cones, placed 2.5 m before and behind the GAITRite system, indicated the start and finish area of the gait assessment, resulting in steady-state measurement over the walkway. Participants walked across the walkway once at their usual gait speed and once at their individual maximum gait speed. The instruction for the walk at usual pace was: “Please walk in your usual gait speed until you reach the indicated cone.” For maximum pace, the instruction was to “walk as quickly and safe as possible without running.”

#### Self-perceptions of aging (SPA)

We used the two standard subscales “SPA physical losses” and “SPA ongoing development” of the AgeCog-Scales [5, 33] to measure SPA. The loss-related subscale “SPA physical losses” refers to the perception of aging as associated with physical decline and decreasing vitality and fitness. An example item is “Aging means to me that I am less healthy”. The gain-related subscale “SPA ongoing development” refers to the notion that aging is associated with making new plans and following ideas. An example item is “Aging means to me that my capabilities are increasing”. Each subscale contains four items on a four-point Likert-scale ranging from 1 “definitely true” to 4 “definitely false”. We recoded the answers for the analysis so that a higher score signifies greater endorsement of the respective SPA domain. Scores were averaged across all four items of the respective subscale. Cronbach’s α was 0.81 for SPA physical losses and Cronbach’s α = 0.71 for SPA ongoing development.

#### Covariates

As covariates, we included the Big Five personality traits (openness, agreeableness, neuroticism, conscientiousness, and extraversion) as assessed with the 10-item short version of the Big Five Inventory [34], since personality traits have been associated with gait (e.g., gait speed [24] and walking limitations [25]) as well as objective physical function [35] in other studies. In addition, personality traits partly shape SPA [36] and both constructs are distal correlates of physical function. We thus controlled for personality to investigate unique associations of SPA with gait patterns. Respondents indicated the extent to which they agreed to the items on a 5-point Likert-scale ranging from 1 (do not agree) to 5 (totally agree). The score for each trait was averaged over the corresponding two items.

We also controlled for number of illnesses as assessed with the Functional Comorbidity Index [37], age, gender (0 = male, 1 = female), and years of education.

As the participants displayed overall good cognitive function with strong ceiling effects in the Montreal Cognitive Assessment, we chose not to include this variable in the analyses.

In addition, global physical function of the participants was assessed with the Short Physical Performance Battery [38] to help to characterize the groups identified by the LPA.

### Analytical procedure

For descriptive analyses, we used IBM SPSS 25. Multivariate outliers were identified using Cook’s distance. Three participants in the normal gait speed condition and one participant in the maximum speed condition had a distance > 1 and were therefore excluded from the analysis.

To identify gait patterns in the sample, we conducted a latent profile analysis (LPA) using Mplus7. The LPA identifies patterns in the data in an explorative manner: it identifies groups that are maximally homogenous within and maximally heterogenous between one another. The researcher must decide on the best fitting amount of patterns based on several indicators of model fit (e.g., AIC, BIC) and theoretical considerations. First, we decided on the gait parameters we used to form gait patterns. Based on the work of Lindemann [15], we chose four gait parameters that each represent an important gait feature: We used gait speed (m/s) to represent walking capacity and the variability of stride-length and step-width (coefficient of variation: CoV = SD/mean (in cm) × 100 [%]) to represent regularity and adaptability of gait. Additionally, we included the walk-ratio (cm/steps/min) to represent the spatiotemporal coordination of walking. The goal was to identify homogenous groups of individuals exhibiting similar gait patterns based on these four parameters. Individuals were assigned to a group based on the maximum posterior probability of group membership derived from the LPA. To further characterize groups in terms of physical function, we used normative values of Beauchet and colleagues [39] as well as a t-test to identify differences in SPPB-Scores.

In a second step, we used IBM SPSS 25 to apply binary logistic regression to the data to test the hypotheses about differential associations of SPA physical losses and SPA ongoing development with gait patterns. This regression tests whether the predictors increase the odds to exhibit a certain gait pattern. In both regression analyses, we added as predictors the two SPA domains of physical losses and ongoing development and as covariates the Big 5 personality traits, age, gender, education and the number of illnesses to predict whether a person exhibits a certain gait pattern.

We checked the predictors for multicollinearity using Pearson’s r and the variance inflation factor (VIF). All correlations were below *r* = 0.408 and VIF was close to 1 which suggests no problems with co-/multicollinearity.

## Results

### Descriptive results

Table 1 shows the sample characteristics of the 150 study participants. Their mean age was 80.45 years (*SD =* 4.46) and 61% were women. They were highly educated with a mean education of 13.65 years (*SD =* 3.27). On average, they associated their aging more strongly with ongoing development (*M =* 3.03, *SD =* 0.68) than physical losses (*M =* 2.85, *SD =* 0.79).

**Table 1:** Means (M), standard deviations (SD) and range of the predictors of gait pattern

*N* = 150	M or %	SD	Range
**Age**	80.45	4.46	71–93
**Female**	61.00		
**Years of education**	13.65	3.27	8–28
**Number of illnesses**	3.59	2.23	0–9
**SPA ongoing development**	3.03	0.68	1.00–4.00
**SPA physical losses**	2.85	0.79	1.00–4.00
**Extraversion**	3.39	1.17	1.00–5.00
**Neuroticism**	2.64	1.12	1.00–5.00
**Openness**	4.03	1.02	1.00–5.00
**Conscientiousness**	4.04	0.95	1.50–5.00
**Agreeableness**	3.46	0.98	1.00–5.00

### Gait patterns

**Fig. 1:**
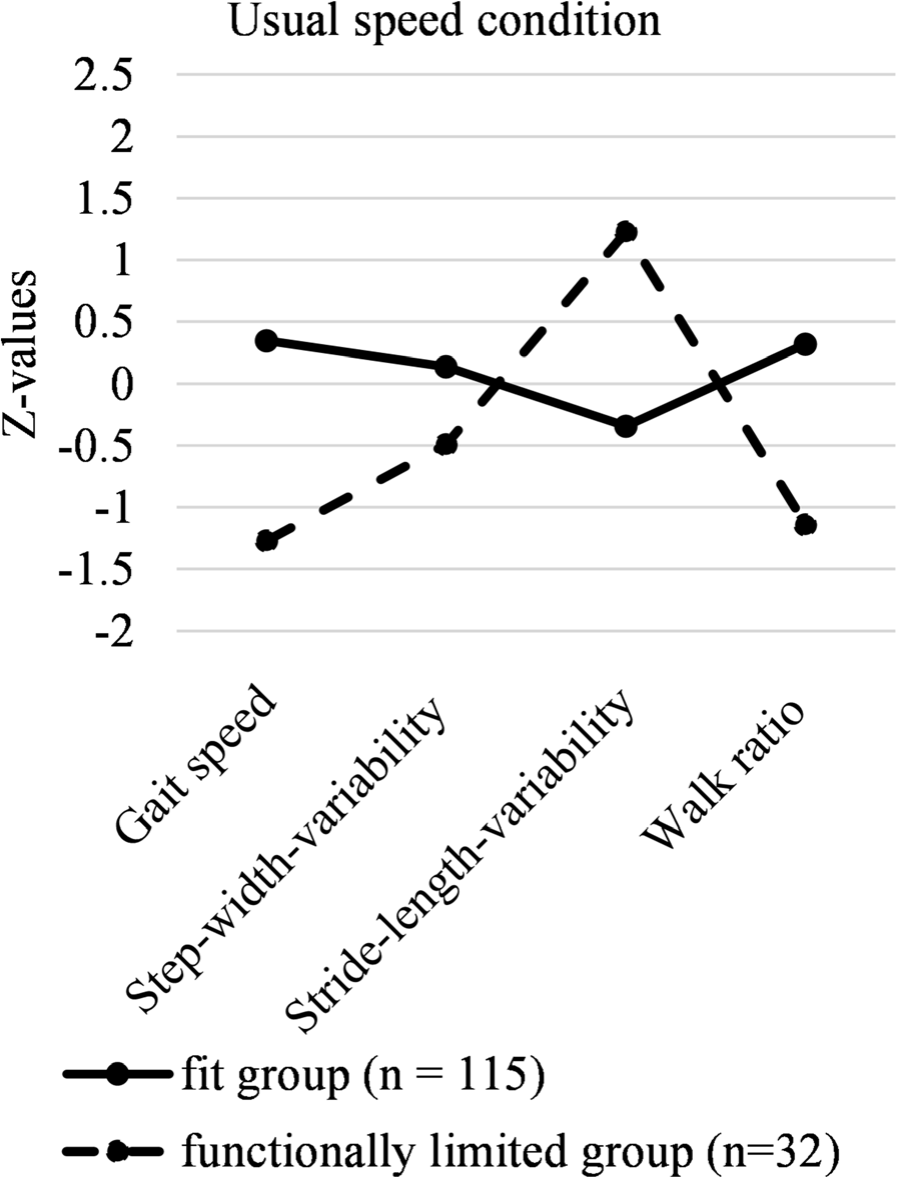
The two gait patterns in the usual gait speed condition. Note: To facilitate the display of the results, we z-standardized the variables for the figures

**Fig. 2:**
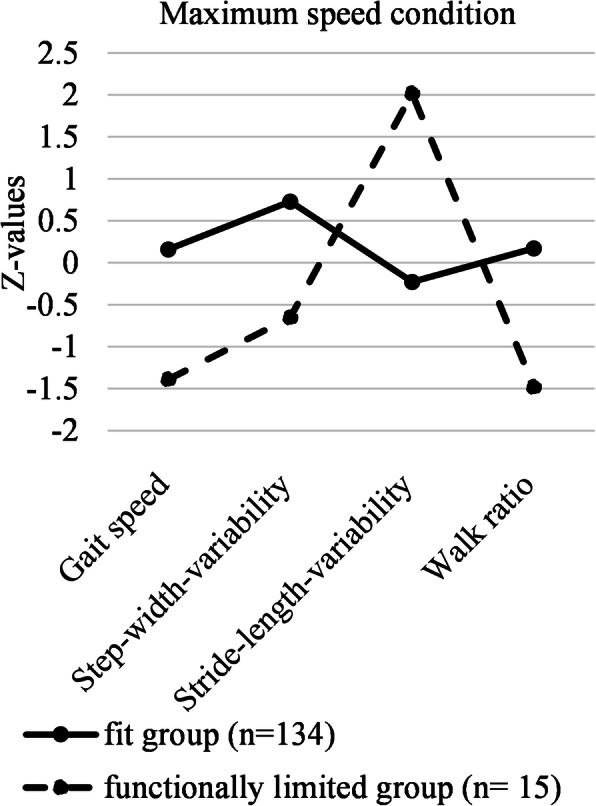
The two gait patterns in the maximum gait speed condition. Note: To facilitate the display of the results, we z-standardized the variables for the figures

LPA was able to distinguish gait patterns in the data. Based on AIC, BIC and the significant parametric bootstrapped likelihood ratio test (BLRT), we decided on a solution with two different gait patterns in both gait speed conditions. These two gait patterns were meaningful on theoretical grounds. The two patterns are represented by the straight and dotted lines in Fig. 1 (usual speed condition) and Fig. 2 (maximum speed condition). In the usual gait speed condition, a large (*n* = 115), relatively “fit” group emerged, represented by the straight line in Fig. 1. This group exhibited a gait pattern with a mean gait speed of 1.30 (*SD* = 0.18), a mean step-width-variability of 29.09 (*SD* = 14.58), a mean stride-length variability of 2.46 (*SD* = 0.91) and a mean walk ratio of 0.58 (*SD* = 0.07). The second, much smaller group (*n* = 32) can be termed “functionally limited”. This group had a mean gait speed of 0.91 (*SD* = 0.15), a mean step-width-variability of 20.16 (*SD* = 9.89), a mean stride-length variability of 4.66 (*SD* = 1.49) and a mean walk ratio of 0.46 (SD = 0.06). The dotted line in Fig. 1 represents this functionally limited group. The average latent class probabilities for most likely latent class membership were 0.922 in the fit group and 0.974 in the functionally limited group.

In the maximum speed condition, also two groups emerged: A large (*n =* 134) fit group had a mean gait speed of 1.58 (*SD =* 0.29), a mean step-width-variability of 28.06 (*SD =* 13.94), a mean stride-length variability of 2.80 (*SD =* 1.32) and a mean walk ratio of 0.55 (*SD =* 0.08). The straight line in Fig. 2 represents this fit group. The smaller (*n =* 15), functionally more limited group had a mean gait speed of 1.05 (*SD =* 0.45), a mean step-width-variability of 17.81 (*SD =* 12.37), a mean stride-length variability of 7.18 (*SD =* 2.31) and a mean walk ratio of 0.40 (*SD =* 0.07). The dotted line in Fig. 2 represents this functionally limited group. The average latent class probabilities for most likely latent class membership were 0.989 in the fit group and 0.893 in the functionally limited group.

### Further analysis

To justify the labelling of the groups as “fit” and “functionally limited”, we compared their SPPB scores in a t-test. In the usual speed condition, the mean SPPB scores were 11.44 (*SD =* 0.91) in the fit group and 9.41 (*SD =* 2.06) in the functionally limited group (*t*(34.43) = − 5.45, *p <* 0.001). In the maximum speed condition, the mean SPPB scores were 11.22 (*SD =* 1.12) in the fit group compared to 8.13 (*SD =* 3.02) in the functionally limited group (t(14.43) = − 3.92, *p ≤* 0.001).

### SPA and gait patterns

To test the hypothesized associations of SPA physical losses and SPA ongoing development with gait patterns, we performed two binary logistic regressions in SPSS 25 (see Table 2). Hypothesis 1 about the association of SPA physical losses with a less favorable gait pattern in the usual speed condition is supported by the data. Higher SPA physical losses increased the odds of belonging to the functionally limited group in this condition (OR = 2.69, 95% CI 1.23–5.85). In addition, participants who were more extraverted (OR = 1.79, 95% CI 1.12–2.86) and those that were older (OR = 1.85, 95% CI 1.15–2.89) had increased odds of belonging to the functionally limited group, whereas gender and education did not reach significance. Number of illnesses was close to significance with OR = 1.30, 95% CI 1.00–1.68).

**Table 2:** Results of the binary logistic regression analyses of gait pattern group on SPA

Predictors	Usual gait speed condition	Maximum gait speed condition
	*OR (95% CI)*	*OR (95% CI)*
**SPA physical losses**	2.69 (1.23–5.85)**	1.40 (0.51–3.85)
**SPA ongoing development**	0.66 (0.29–1.15)	0.38 (0.15–0.98)*
**Extraversion**	1.85 (1.15–2.89)*	1.31 (0.73–2.35)
**Neuroticism**	0.88 (0.53–1.45)	0.60 (0.32–1.12)
**Openness**	1.48 (0.79–2.77)	1.46 (0.70–3.05)
**Conscientiousness**	0.73 (0.40–1.32)	0.88 (0.45–1.70)
**Agreeableness**	1.08 (0.61–1.93)	0.72 (0.35–1.47)
**Age**	1.29 (1.14–1.47)***	1.14 (0.99–1.31)
**Gender**	2.37 (0.63–8.93)	2.94 (0.55–14.69)
**Education (years)**	1.12 (0.93–1.35)	1.06 (0.86–1.32)
**No. of illnesses**	1.30 (1.00–1.68)	1.45 (1.04–2.04)*

Note: Reference group: fit group. Values above 1 imply an increased likelihood of belonging to the functionally limited group. Values below 1 imply an increased likelihood of belonging to the fit group

*p < 0.05; **p < 0.01; ***p ≤ 0.001

Hypothesis 2 about the association of SPA ongoing development with a more favorable gait pattern in the maximum speed condition is also supported: SPA ongoing development decreased the odds of belonging to the functionally limited group (OR = 0.38, 95% CI 0.15–0.98). In addition, the number of illnesses increased the odds of belonging to the functionally limited group (OR = 1.45, 95% CI 1.04–2.04) in the maximum speed condition.

Taken together, binary logistic regression analyses provided support for both hypotheses on the differential effects of the two SPA domains for gait patterns under two gait speed conditions.

## Discussion

In this study, we wanted to add to the scarce literature on associations of SPA with objectively measured gait parameters as an indicator of physical function in older adults. Extending previous studies, we investigated differential associations of two domains of SPA with gait patterns: SPA as associated with physical losses and SPA as associated with ongoing development. Furthermore, we did not use single gait parameters but instead accounted for the complex nature of gait by using gait patterns as our outcome. To our knowledge, this study is the first to explore gait patterns based on LPA with this selection of gait parameters in a community sample of older adults.

### LPA as an approach to identify gait patterns

Based on four objectively measured gait parameters that each represent a different gait feature [15], we found two distinct gait patterns—a fit one and one that reflected functional limitations—in the usual as well as the maximum speed condition. The means of all gait parameters in the fit group were above normative values based on usual gait speed [39], while means of all gait parameters in the functionally limited group were below these normative values. This finding supports our differentiation of the groups in fit vs. functionally limited, which is further supported by significantly differing SPPB-scores between the groups in both conditions. However, our approach goes beyond comparing single means of gait parameters between pre-defined groups; the explorative LPA looks for patterns in the data that differentiate homogenous subgroups based on *certain combinations* of gait parameters. This approach accounts for the complexity of gait.

### Differential associations of SPA with gait patterns

In addition, the present study considered gain- and loss-related SPA in the domains of physical losses and ongoing development and thus used a multidimensional and multidirectional approach to SPA. Previous studies often used a global, unidimensional measure of SPA. Like examination of a single gait parameter, this yields valuable insights, yet needs further differentiation to fully understand associations and implications. For this reason, we tested specific hypotheses on associations of loss-related SPA in the domain of physical decline and gain-related SPA in the domain of ongoing personal development with gait patterns. In line with research on SPA and self-reported physical function [11], we found that SPA physical losses were associated with a higher likelihood of exhibiting an objectively measured, functionally limited gait pattern in the regular speed condition: those belonging to the functionally limited group perceived aging more strongly as associated with physical losses than those in the fitter group. In previous studies, this SPA domain was also associated with worse self-reported physical and mental health [40] and a higher number of self-reported physical illnesses [5]. This suggests that people with SPA physical losses indeed encounter physical limitations more often in their everyday lives, which is reflected in a functionally limited gait pattern. However, this study cannot determine causality; it might as well be the case that the experience of physical decline leads some people to think about their own age and aging as associated with physical losses.

We also investigated the role of SPA ongoing development for gait patterns. Previous studies found that positive SPA is associated with positive health outcomes [1, 5]. The present study’s findings point into the same direction. For the gain-related SPA ongoing development, we found that this SPA domain increased the odds of belonging to the fit group in the maximum speed condition. This means, SPA ongoing development were associated with better gait performance in a condition that requires physical effort: Individuals with more SPA ongoing development showed a more stable, better coordinated and faster gait performance. This finding is remarkable since it provides further evidence that a positive view on one’s own aging can be reflected in a more stable gait pattern and the ability to mobilize physical resources in old age. Yet, also here we cannot determine causality. The findings might as well show that a person who is able to walk stable at maximum speed possesses resources and energy that are reflected in gain-related SPA ongoing development.

Not only did our findings support previous findings showing associations of positive and, more specifically, gain-related SPA with a variety of positive health variables [4]; we were also able to show that there are domain-specific associations of SPA with gait performance under different walking conditions.

### Strengths and limitations

This study has several limitations. One major limitation is the risk that some of our chosen gait parameters may not be reliable: We included the variabilities of step-length and step-width based on only a few gait cycles. However, according to Hollman, Childs et al. (2010), [41], a good reliability of variabilities requires more strides than we assessed in our study. This might have affected the accuracy of the LPA, and the regressions that follow from these. Furthermore, we had a relatively small sample that was selective in terms of health and education—the participants had relatively good physical function and reported an above average education. Accordingly, the number of individuals exhibiting the functionally limited gait pattern was rather small compared to the group with the fit gait pattern. In addition, our cross-sectional design does not allow for causal interpretation of effects. Furthermore, it has to be noted that conducting the LPA in Mplus and exporting the estimated class membership to SPSS has the potential for both rounding errors and non-equivalent estimation, which might have affected the results. However, our study also has several strengths. On a methodical level, we showed that LPA can be a useful approach to differentiate gait patterns. We also expanded past findings on associations of SPA and self-reported physical function by adopting an objective measure of physical function. Furthermore, we investigated differential effects of two domains of SPA—most other studies used a unidimensional positive/negative measure for SPA. On the one hand, our results strengthen previous findings on the role of SPA for (self-reported) physical function, thereby stressing the importance of SPA as a health-relevant concept. On the other hand, our approach also adds to the literature on gait, since we introduced SPA as a relevant construct for research on gait patterns, which could benefit from acknowledging the importance of distal psychological variables other than personality for gait performance.

### Implications for future research

Our study showed that latent profile analysis is a promising approach to identify gait patterns not only for groups with specific illnesses [18], but also in community samples of older adults. Future research could use this method in larger, more diverse samples to identify a higher number of more differentiated gait patterns. This would allow for tailoring of interventions based on specific needs of the identified subgroups. In addition, future research is needed to confirm our findings. Especially, these studies should be based on longer walks to overcome issues of low reliability of the variability parameters [42]. Future studies should also include a larger sample size and could additionally select gait parameters based on other approaches, e.g. Hollman, McDade et al. [43]. Furthermore, our findings show that SPA are not only important in the context of self-views of physical function, as represented by self-report data, but also in the context of objectively measured gait performance as an indicator of physical function. Especially the finding that SPA ongoing development are associated with the mobilization of physical resources in the maximum speed condition points to the importance of a positive view on aging as a source of reserve capacity for physical function in later life; and it points to the importance of good physical function for gain-related SPA.

Longitudinal findings on SPA and self-reported health suggest that SPA work as a self-fulfilling prophecy. This means, those who view aging as associated with physical losses are more likely to actually experience physical losses; in contrast, those associating aging with personal development are more likely to have positive experiences [4]. Future studies should thus investigate longitudinal associations of domain-specific SPA with objectively measured gait to corroborate the cross-sectional findings of our study and to infer direction of causality. Furthermore, it might be interesting to investigate whether associations of SPA with gait are mediated by proximal factors (e.g., fear of falling) and to investigate the role of conscious movement processing inclination in the context of SPA and gait.

In a next step, interventions to improve physical function in older adults should more often address the SPA of the participants and promote a positive view on aging. There is first evidence that the combination of physical exercise and an intervention on SPA is a promising approach [34]. Interventions on physical activity might additionally provide a good basis to sharpen older adults’ eyes for gains and positive experiences in later life, which can be a source for happiness and comfort regardless of age. This view, in turn, might have a positive impact on their physical function.

## Conclusions

As a conclusion, studies that explore the gait of older adults should more often include SPA, as these views are not only related to subjective measures of physical function, but also to objective physical function as assessed with gait patterns. Furthermore, we were able to show domain-specific associations of SPA on physical function: those who viewed their aging as associated with physical losses were more likely to exhibit physical limitations at regular speed. However, those who perceived their aging as associated with ongoing psychological development were more likely to keep up a stable, fast gait at maximum speed. Our findings add to the literature on SPA and physical function and should be further explored in longitudinal studies.

## Data Availability

The datasets generated during this study are not publicly available. However, they are available from the corresponding author on reasonable request.

## Abbreviations

LPA: Latent profile analysis
SPA: Self-perceptions of aging
